# Plant-Based Gums and Mucilages Applications in Pharmacology and Nanomedicine: A Review

**DOI:** 10.3390/molecules26061770

**Published:** 2021-03-22

**Authors:** Mohammad Sadegh Amiri, Vahideh Mohammadzadeh, Mohammad Ehsan Taghavizadeh Yazdi, Mahmood Barani, Abbas Rahdar, George Z. Kyzas

**Affiliations:** 1 Department of Biology, Payame Noor University, Tehran 19395-4697, Iran; amiriherb@gmail.com; 2 Department of Pharmaceutical Nanotechnology, School of Pharmacy, Mashhad University of Medical Sciences, Mashhad 917794-8954, Iran; MohammadzadehV971@mums.ac.ir; 3 Applied Biomedical Research Center, Mashhad University of Medical Sciences, Mashhad 917794-8564, Iran; metyazdi@gmail.com; 4 Department of Chemistry, Shahid Bahonar University of Kerman, Kerman 76169-14111, Iran; mahmoodbarani7@gmail.com; 5 Department of Physics, University of Zabol, Zabol 98613-35856, Iran; 6 Department of Chemistry, International Hellenic University, 65404 Kavala, Greece

**Keywords:** gum, mucilage, nanomedicine, herbal medicine, pharmacology

## Abstract

Gums are carbohydrate biomolecules that have the potential to bind water and form gels. Gums are regularly linked with proteins and minerals in their construction. Gums have several forms, such as mucilage gums, seed gums, exudate gums, etc. Plant gums are one of the most important gums because of their bioavailability. Plant-derived gums have been used by humans since ancient times for numerous applications. The main features that make them appropriate for use in different applications are high stabilization, viscosity, adhesive property, emulsification action, and surface-active activity. In many pharmaceutical formulations, plant-based gums and mucilages are the key ingredients due to their bioavailability, widespread accessibility, non-toxicity, and reasonable prices. These compete with many polymeric materials for use as different pharmaceuticals in today’s time and have created a significant achievement from being an excipient to innovative drug carriers. In particular, scientists and pharmacy industries around the world have been drawn to uncover the secret potential of plant-based gums and mucilages through a deeper understanding of their physicochemical characteristics and the development of safety profile information. This innovative unique class of drug products, useful in advanced drug delivery applications, gene therapy, and biosynthesis, has been developed by modification of plant-based gums and mucilages. In this review, both fundamental and novel medicinal aspects of plant-based gums and mucilages, along with their capacity for pharmacology and nanomedicine, were demonstrated.

## 1. Introduction

Extensive use of various excipients, such as binders, thickening agents, sweeteners, and glidants, which can change the physicochemical properties of the final formulation of the drug and adjust the pharmacodynamic and pharmacokinetic properties, has made significant progress in the field of drug delivery systems [1]. Polymers are used as excipients for the progress of polymer-based drug delivery systems with the purpose of targeted drug delivery [2,3,4,5,6,7,8,9]. Synthetic polymers have high physical, chemical, and mechanical stability but can cause cytotoxicity and are bio-incompatible [10]. Synthetic polymers have disadvantages, such as: poor adaptation to the patient’s body, high cost, and can also cause acute and chronic side effects, for example: poly-(methyl methacrylate) (PMMA) can cause skin and eye irritation; povidone accumulates in the limbs at the injection site during subcutaneous injection and forms granulomas; animal studies have shown that carbomer-934P is toxic in oral consumption and the resulting dust has also caused allergic reactions in the eyes, mucous membranes and respiratory tract; and the use of polyvinyl alcohol aqueous solution in subcutaneous injection has also caused anemia. Other disadvantages of synthetic polymers used in tissue engineering include: low biocompatibility, release of acidic products during degradation that can cause systemic and local reactions, and rapid loss of mechanical strength [11].

Recently, the utilization of natural polymers has increased [12,13,14,15,16,17,18]. The use of natural plant-derived polysaccharides as excipients has increased in the pharmaceutical industry and can solve formulation problems and reduce the side effects of synthetic polymers [19,20]. Natural polysaccharides are formed by their *O*-glycosidic linkages by binding monosaccharide residues together and are known as biopolymers [21]. Gums and mucilages are among these excipients. They are widely used in the medicine and cosmetic industries and can also be modified for use in a variety of drug delivery systems [22]. These materials can be used in several pharmacological forms, for instance, control release systems, film-coating agents, nanoparticles, viscous liquid formulations such as ophthalmic solutions, suspensions, implants, etc. Gums and mucilages are composed of many compounds, including polysaccharides. The resins and tannins in the gums are responsible for providing and delivering retardant properties to the dosage form and transmitting release inhibitory properties.

Gums come from different parts of plants. The source of some gums may be the seed epidermis, or they may be extracted from the leaves and bark of plants [23]. Gums are considered pathological substances and are caused by damage to the plant or disapproving conditions, such as breaking the cell wall. Acacia tragacanth and guar gum are samples of gums; gums dissolve easily in water [11].

Mucilages are natural products of metabolism and are formed in the cell and do not dissolve easily in water. Mucilages are found in diverse parts of the plant. Mucilage is a thick, sticky substance produced by almost all plants and some microorganisms. Gums and mucilages have certain similarities; both are plant hydrocolloids. They are also a mixture of clear amorphous polymers and monosaccharide polymers and are combined with uronic acid. Gums and mucilages contain hydrophilic molecules that can combine with water to form viscous or gel-like solutions. Advantages of using gums and mucilages in the pharmaceutical industry include: that they are biodegradable, biocompatible, non-toxic, provide better tolerance to the patient and have fewer side effects, do not cause allergies in humans, do not irritate the skin or eyes, and have low production costs [11,24]. However, the use of these materials is limited due to a series of disadvantages. They may have microbial contamination because the moisture content of gums and mucilages is typically 10% higher, and they are carbohydrate in structure. The amount of hydration in them may not be controlled. Additionally, in storage, their viscosity decreases due to contact with water. To eliminate these disadvantages and reduce the limitations, we can use nanosystems in drug delivery. Natural and synthetic nanoparticles, such as liposomes, nanoparticles, and micelles, improve the stability and bioavailability, as well as the biological distribution of natural products. Nanosystems are able to deliver drugs to specific areas, so they can increase local drug concentrations and significantly reduce the adverse effects of drug uptake [25,26,27,28,29,30]. Therefore, natural nanoformulations have attracted a lot of attention [11,31].

This review investigated the pharmacological properties and different applications of plant-based gums and mucilages in pharmaceutical and nanomedicine formulations.

## 2. Methodology

Scientific databases, including Scopus, ISI Web of knowledge, Pubmed, and Google scholar, were searched using the terms “gums” AND “mucilages” etc. In this manuscript, scientific and author names of plant species were determined according to international authority plant database, The Plant List (http://www.theplantlist.org) (accessed 10 March 2021).

## 3. Chemical Character of Gums and Mucilages

Gums and mucilages are polysaccharides, converted to monosaccharides by hydrolysis. The types of hydrolysis products can include pentosan (e.g., xylan) and hexose (e.g., starch and cellulose). Gums contain the salts of potassium, calcium, and magnesium, known as “polyuronides”. Mucilages are sulfuric acid esters; the ester group is a complex polysaccharide. The sugars in gums and mucilages are galactose and arabinose. Identification of sugar-forming units in a polysaccharide by hydrolysis is performed by various chromatographic techniques. The total amount of carbohydrates in a polysaccharide, as well as the content of monosaccharides, can be determined by the phenol-sulfuric acid manner. Also, NMR and mass spectrometry techniques are utilized for structural identification of gums and mucilages [22].

### Classification of Gums and Mucilages

According to their origins, gums and mucilages are found in large amounts in a varieties of land-plant sources (e.g., gum tragacanth, gum arabica, gum ghatti, and karaya gum), animal sources (e.g., hyaluronic acid, chitin and chitosan, and chondroitin sulfate.), marine origin/red seaweeds (e.g., agar and carrageenan) and brown algae sources (e.g., alginate and laminarin), and fungi and other microbial sources (e.g., xanthan, dextran, curdian, pullulan, zanflo, emulsan, schizophyllan, lentinan, krestin, scleroglucan, and Baker’s yeast glycan.), where they perform many structural and pharmaceutical applications. Among them, plant sources provide the largest amounts [32,33].

## 4. Plant-Based Gums and Pharmaceutical Applications

Plant-derived gums are the polysaccharides formed from different parts of the plant (Table 1). Gum tragacanth is one of the most common gums, which has been applied medicinally for many years, with written confirmation of its uses defined by Theophrastus in the 3rd century B.C.

Some tragacanthic species of the genus *Astragalus* L. (Fabaceae) earned dignity owing to their potential in generating gum tragacanth, which has a broad spectrum of usages in drug and various industries. Among them, *Astragalus* gummifer Labill., *Astragalus microcephalus* Willd., *Astragalus brachycalyx* Fisch. ex Boiss., *Astragalus myriacanthus* Boiss., *Astragalus gossypinus* Fisch. and *Astragalus kurdicus* Boiss. are the main species to resource the gum tragacanth in the universal market. In Iran, the tragacanth gum, popularly recognized as “Katira”, has been widely applied in medication and confectionery since ancient periods. In Iranian folk medicine, it is largely used as an analgesic, general tonic, laxative factor, and to cure cough and lip fissures [88]. Herbal gums have been widely used in the pharmaceutical sciences for a variety of applications as stabilizing, binding, suspending, emulsifying, and thickening agents and for the sustained release of drugs [89].

### 4.1. Use of Gums in Medicinal Formulations

In one study, PAGE and *Prunus domestica* L. (Rosaceae) gum were compared with hydroxypropyl methylcellulose (HPMC), and the ability of stable diffusion was investigated in both groups. The results displayed that, when PAGE and *Prunus domestica* gum were employed in a 1:1 combination ratio, the release efficiencies improved, and in the optimal formulation, the diffusion profile was comparable to the standard market formulation, and PAGE could be utilized as a matrix in tablet formulations [89]. In another study, the synergistic binding possibility of PAGE and *Prunus domestica* gum in tablet formulation was examined. The results showed that the gums used had a superior binding ability to prepare the dosage form of the uncoated tablet from PVP K30. In another study, the characteristics of gum bonding were compared with gum arabic and polyvinyl pyrrolidone. The results showed that PAGE is a hopeful drug in tablet formulations [90,91,92].

### 4.2. Use of Gums to Improve Metformin Microspheres

In the study of H. Ozoude et al., in 2020, the formulation and improvement of metformin microspheres by using *Khaya senegalensis* (Desv.) A.Juss. gum as a copolymer were investigated. Khaya gum is a bark secretion from *Khaya senegalensis* (Maliaceae) that is able to carry the drug. The aim of this review was to formulate and compare metformin-loaded microspheres formed with a mixture of khaya gum and sodium alginate. Spherical microspheres with different sizes (1200 to 1420 μm) were acquired. FTIR analysis displayed no important interaction among pure metformin hydrochloride and excipients. The efficiency of drug trapping in the microspheres ranged from 65.6 to 81.5%. Drug secretion from the microspheres was sustained for 9 h of study. A mixture of *Khaya senegalensis* gum and sodium alginate was a favorable polymer composition for formulation with controlled release. The 2:3 formulation ratio containing Khaya gum and sodium alginate, respectively, produced microspheres with controlled release sketches comparable to the trading metformin tablets [93].

### 4.3. Use of Gums as a Drug Carrier to Form Hydrogels and Improve Pharmacokinetics

In a 2020 study, Singh et al. used dietary tragacanth gum (TG) to form hydrogels as drug carriers to improve the pharmacokinetic defect of the anticancer drug methotrexate. Polymer properties were measured by XRD, FTIR, and SEMs techniques. The drug release profile was determined by evaluating some properties, such as blood compatibility, mucosal adhesion, and mechanical strength. The diffusion profile was found using a non-Fickian diffusion process and was the best in Higuchi kinetic model. The results showed that the polymer matrix was non-thrombogenic, compatible with homo, and mucoadhesive. Hydrogels adhere to the intestinal mucosa with a 14.3 ± 4.5 mM adhesion test [94]. In another study by Sharma et al., a penetrating polymer network (IPN) hydrogel based upon acacia gum, AAm-IPN-AA (acrylamide and Ga-cl-poly acrylic acid), was created using a two-step aqueous polymerization. One-step impregnation of silver nanoparticles was performed. The synthesized hydrogels were assayed using FTIR, SEM, and PXRD methods. Silver nanoparticles (AgNPs) in the range of 20–80 nm were fabricated by reduction of silver nitrate by *Koelreuteria paniculata* Laxm. (Sapindaceae) flower extract. The synthesized hydrogels were used as a model for AgNP saturation. To find the characterization of these nanocomposite hydrogels, PXRD, FTIR, and SEM were performed. Preparation of samples with diverse bacterial strains (*Staphylococcus aureus*, *Escherichia coli*, *Pseudomonas aeruginosa*) and fungal strains (*Aspergillus* and *Penicillium*) were exposed to antibacterial/antifungal investigations. The results showed that synthesized nanocomposite hydrogels with anti-bacterial activities reduced bacterial and fungal activities [95].

### 4.4. Investigation of Antibacterial Properties of a Mixture of Polymers and Guar Gums

In another study, a mixture of chitosan/poly(vinyl alcohol)/guar gum (CS/PVA/GG) was organized. The ratio of swelling, together with antimicrobial properties, was studied. SEM results showed that surface morphology was more affected by mixing and bonding ratios. XRD confirmed the crystal structure of the compounds, as did FTIR, a strong intermolecular bond between the polymers. The arranged mixtures displayed good antimicrobial properties against bacterial agents *P. multocida*, *S*. *aureus*, *E. coli,* and *B. subtilis* [96].

### 4.5. Establishing an Oral Delivery System of Protein Drugs by Gums

In the study of A.R. Freitas et al., the oral delivery system for protein drugs was evaluated by *Sterculia striata* A. St.-Hil. and Naudin (Malvaceae) gum. Natural polysaccharides were tested as carriers for oral insulin administration. Due to their non-toxicity, degradability, and cheap and easy availability, gums have many uses in the pharmacological production. This work aimed to create a gum-based formulation of *Sterculia striata* with extra bio-polymers (dextran sulfate, chitosan, and albumin), a cross-linking factor (calcium chloride), and stabilizing agents (polyethylene glycol and polyxamer 188) to enhance bio-availability. Insulin was employed as a drug pattern and the ways used to prepare the formulation were on the basis of ionotropic precursors and, subsequently, electrolytic complexes of biopolymers with opposite charge under pH-controlled conditions. A formula was developed to determine its effectiveness by determining its mean particle size (622 nm), insulin encapsulation efficacy (70%), constancy (in storage for 30 days), and adhesion strength in vitro (92.46 mM). It was found, in addition, the developed formulation retained about 64% of the early insulin dosage in a pretended gastric environment. This research, for the first time, performed an insulin delivery system based on *Sterculia striata* gum with a high possibility for oral administration of protein drugs, which is a valid option for effective delivery of those drugs [97].

## 5. Plant-Based Mucilages and Pharmaceutical Applications

Several species of mucilaginous plants have been applied in diverse traditional medical systems throughout the world for more than 4000 years [98]. Currently, in pharmaceutical formulations, diverse mucilages have been employed as a binding factor and a drug excipient. Mucilage has good bonding activities compared to numerous synthetic materials [22,99,100,101,102,103,104]. In general, the use of mucilages in drug formulations includes their usage in the production of tablets [105,106], as an emulsifying and suspending agent [22,107,108], as a bioadhesive agent [109], as well as gelling and thickening agents [110,111]. In inflammatory processes of the gastrointestinal tract, mucilages have been used in medicinal formulations. The mechanism of action of a mucilage is that it covers the mucous membranes and prevents stimulation of the nerve endings (8).

### 5.1. Use of Mucilages as an Adjunct and Suspending Factor in Medicinal Formulations

A 2020 study by Haile et al. examined the physicochemical properties of *Grewia ferruginea* Hochst. ex A.Rich. (Malvaceae). Mucilage is potentially rich as a medicinal excipient. The aim of this study was to describe the bark mucilage of *Grewia ferruginea* for its possible usage as a medicinal drug. Mucilage was extracted by extracting water from the bark of the inner stem of *Grewia ferruginea*, precipitated with ethanol, desiccated and powdered. Powdered mucilage was identified for various physic–chemical assets, for instance, powder properties, drying loss, solubility and swelling index, ash content, pH, viscosity, moisture absorption, microbial load, and acute oral toxicity. As stated by the results, the final yield percentage of dried and powdered GFM was 11.96% (*w*/*w*). Compaction attributes showed good powder flow properties. GFM displayed false current behavior. Moisture absorption of GFM showed the nature of its moisture, and its solubility and swelling improved with temperature. The pH of GFM was almost neutral. The microbial load of mucilage was pharmacological, and acute oral toxicity testing showed that mucus is safe up to 2000 mg/kg. From the results of this study, it could be concluded that *Grewia ferruginea* bark mucilage can be used as an adjunct in medicinal formulations [112].

In the study of Sibhat et al., a study was conducted to evaluate the effect of *Grewia ferruginea* (GFM) mucilage as a suspending factor on a metronidazole benzoate suspension. Suspensions were set up with 0.5, 1, 1.5, and 2 percentages of GFM and compared to suspensions prepared from XGM (xanthan gum) and SCMC (sodium carboxymethylcellulose) at similar concentrations. The prepared suspensions were assessed in terms of visual appearance, pH, rheology, sediment volume and decomposability, degree of clotting, and drug release characteristics in the laboratory. The stability study was performed for three months in different storage conditions. The results showed that the entirety of prepared suspension formulations showed the properties of quasi-plastic flow with viscosity transfer capability of the suspension agents, respectively. Flow rate and reproducibility of formulations prepared with GFM were meaningfully less than those with SCMC and higher than those with XGM. At 0.5% concentration of the suspending agent, the deposition volumes of the formulations were, respectively. Though, at all other concentrations, the deposition volume of the GFM-prepared formulation had like consequences to the XGM but showed a meaningfully higher deposition volume than the SCMC. Formulations with GFM showed an upper grade of clotting at a concentration of 0.5% but were comparable at 1.5% with formulations containing XGM. The pH, examined, and in vitro release profile of all formulations evaluated were pharmacological. Therefore, based on the findings of this investigation, it can be determined that the mucilage of the bark of *Grewia ferruginea* could be used as a suspending factor in suspension formulations [113].

### 5.2. Use of Mucilages to Create Porous Physical Structures and Cell Scaffolds

**Figure 1:**
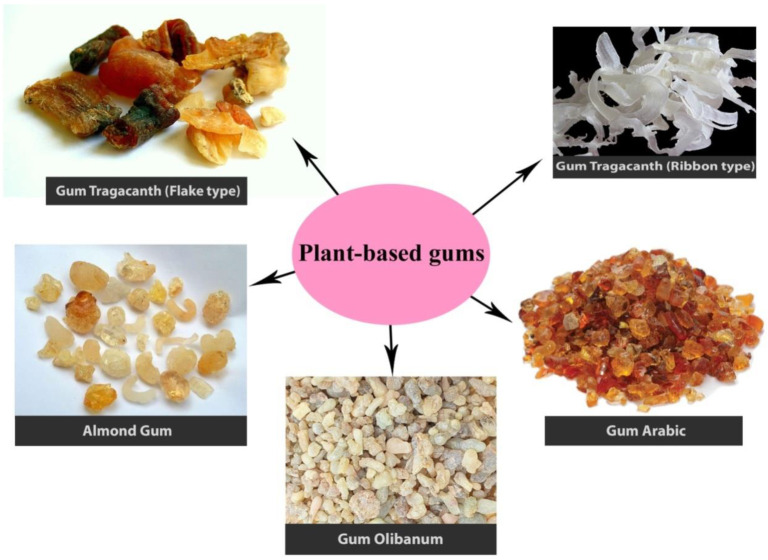
Some of the most important gums used in the world.

In another study, quince seed mucilage was used to make a porous physical structure for medicinal applications. Quince seed mucilage was extracted, molded, and dried frozen. Scaffolds derived from interconnected seed mussel had a completely interconnected porous construction. Subsequently, human-adipose-derived mesenchymal stem cells were seeded on the crosslinked quince seed-mucilage-derived scaffolds, and cell viability in scaffolds was evaluated by 3-(4,5-dimethylthiazol-2-yl)-2,5-diphenyl-2H-tetrazolium bromide (MTT) assay. MTT results showed that scaffolds had no cytotoxic effect on granular cells. Adhesion and migration of human-adipose-derived mesenchymal stem cells on scaffolds derived from interconnected seed mucilage, moreover, were assessed histologically by hematoxylin and eosin staining by scanning electron microscopy analysis. As a result, transplanted seed-mucilage-derived scaffolds have the potential to replace common polysaccharides in regenerative medical applications [114]. Figure 1 shows some of the most important gums used in the world.

Some of the most important mucilaginous plant species, along with their pharmaceutical uses, are indicated in Table 2.

## 6. Importance of Plants in the Biosynthesis of Nanoparticles

**Figure 2:**
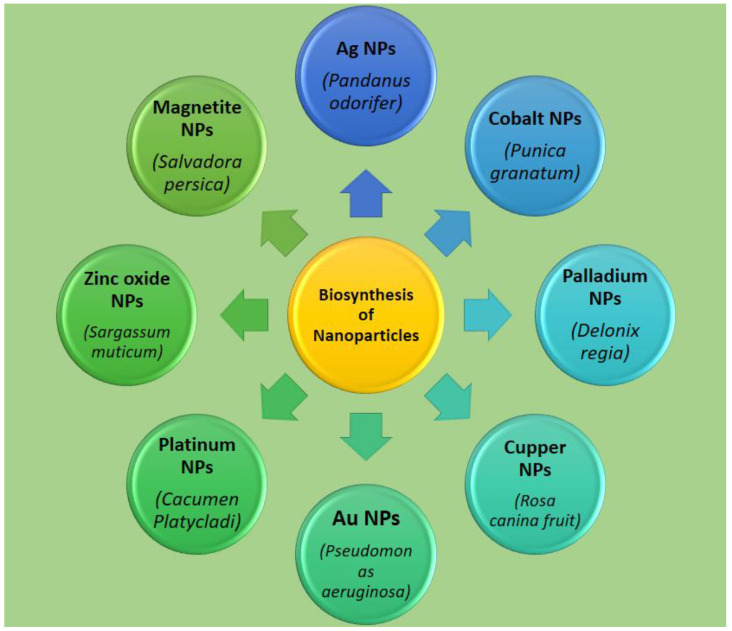
Various nanoparticles derived from plant resources, extract presented in the bracket.

Various macro or microscopic species, such as plants, fungi, bacteria, seaweed, and microorganisms, can carry out the biological synthesis of nanoparticles [124,125,126]. Until now, different diseases have been successfully handled by bio-synthesized nanomaterials with fewer toxic consequences [127]. Plentiful natural products, such as flavonoids, steroids, alkaloids, saponins, tannins, and other dietary materials, are present in plants [128]. These bioactive compounds are extracted from different parts of plants (flowers, roots, leaves, seeds, root shoots, and barks). Numerous researchers have clearly shown that plant extracts serve as a possible precursor to nanoparticle synthesis in a non-hazardous situation [129]. Due to numerous secondary metabolites present in plant extracts, the extract acts as a bio-reduction compound and a stabilizing agent for the synthesis of novel metallic nanomaterials [130,131]. Non-biological techniques, such as chemical and physical approaches that are used for synthesizing nanoparticles, are seriously hazardous and highly toxic to biological entities. Furthermore, the natural synthesis of nanoparticles is a cost-effective, single-step, and environmentally safe approach [124]. Plant extract is successfully used in the fabrication of different environmentally friendly nanoparticles, such as silver, copper, platinum, magnetite, cobalt, gold, palladium, and zinc oxide (See Figure 2). Plant-mediated nanoparticles are also possible treatments for HIV, infectious illnesses, malaria, hepatitis, cancer, and other severe diseases [132,133,134].

**Figure 3:**
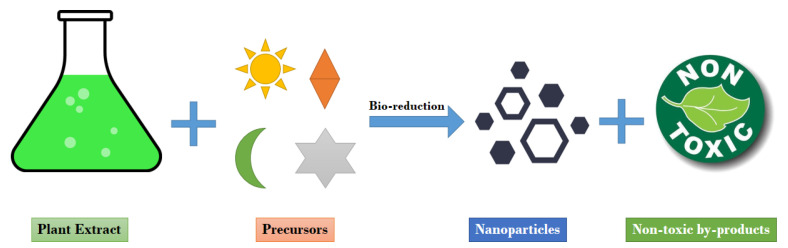
A suggested scheme for nanoparticle synthesis by plant extracts.

The factors that affect the synthesis of plant-based nanoparticles can be challenging [135], for example, different concentrations of hydrogen ions can change the shapes and sizes of the resulted nanoparticles [136]. On the other hand, temperature is another affecting factor for the green synthesis of nanoparticles with various sizes and structures [137,138]. Additionally, certain external conditions such as chemical and physical parameters regulate the crystalloid structure of the nanoparticles (NPs) [139]. One of the reasons for reducing the ions molecules into NPs with variant structures is the declining reaction time. To classify the higher concentration of NPs in the medium, the optimal time generates a larger absorption peak value. Finally, with the approach of controlling the growth conditions of synthesis, various shapes of NPs, such as circular, rectangular, triangle, and octagonal, can be achieved [124,140,141]. Figure 3 demonstrates the schematic process of nanoparticle synthesis via plant extracts.

## 7. Applications

### 7.1. Applications of Plant-Derived Gums in Nanomedicine

Natural gums are complex carbs or polysaccharides comprising one or more types of components of monosaccharides, or their equivalents, joined together to build a macromolecular framework with a myriad of connections and architectures. Many simple sugars, such as galactose, mannose, arabinose, glucose, xylose, and uronic acids, are formed by hydrolysis [23,62,142].

Thanks to high biodegradability, adequate availability, non-toxicity, and low-price synthesis, gums are the essential components in many bioactive compounds. Gums compete with other synthetic materials for use as pharmaceutical products these days and have had a great adventure from being a precursor for the synthesis to becoming a revolutionary nanocarrier [22,143,144]. Specifically, scientists and drug companies around the world have discovered the high potential of natural gums by a deeper understanding of their physical and chemical properties [116,145]. Also, modification of natural gum has created a new class of polymers that are useful in developed drug delivery systems [146,147,148,149,150,151,152]. With these modifications, natural gum has expanded its applications as novel drug delivery systems in nanomedicine and gene delivery [22,153,154].

Gums derived plants are used as a stabilizer in many nano-pharmaceuticals [155,156]. Metal nanoparticles (Au and Ag NPs) that are covered with suitable stabilizers can provide stabilized NPs against accumulation and keep them stable in acid and alkaline environments [157,158,159]. Inorganic NPs can be stabilized by a natural gum in two ways: first, by adsorption to the surface of NPs, which causes steric repulsion between the NPs; and second, by increasing the viscosity of the suspension of NPs and, thus, slowing down the accumulation of particles [160]. It has been shown that gums with a plant basis, such as gum acacia, can be used as reductant and stabilizer agents for the biosynthesis of Ag NPs [161]. Pooja et al. evaluated the potential of xanthan gum (XG) in Au NPs biosynthesis as both a stabilizing and reducing agent [162]. In the pH range around pH 5 to 9 and NaCl concentrations up to 0.5 M, plant-based NPs were stable. Also, up to 24 h, nanoparticles displayed considerable stability in serum.

Another interesting application of gum in nanomedicine is gene delivery [163,164]. Polymeric vectors are a pioneering class of gene carriers among non-viral vectors engineered for the safe delivery of genes to target sites because of many benefits, such as safety, cost-effectivity, lower toxicity, and the ability to deliver larger genes [165]. In particular, polyethyleneimine (PEI), with a size of 25 kDa, is a great vector candidate for its pretty high rate of transfection in a variety of organs. However, the primary cause of its marked toxicity appears to be a very high positive charge density on PEI, therefore, limiting its use as an in vivo gene delivery vector [166,167]. For the partial inactivation of its extra positive charge, natural gums containing anionic groups are used to bypass PEI toxic effects. This helps to improve the efficacy of the transfection by reducing its positive charge. For example, Goyal et al. prepared a branched polyethyleneimine and mixed it with gellan gum, an anionic heteropolysaccharide, to create gellan gum-polyethyleneimine (GP) nanocomposites for partial neutralization of its extra positive charge. Findings of in vivo gene expression in Balb/c mice showed maximum luciferase enzyme expression in the spleen. The current study suggested that, with various biomedical applications, GP can act as an effective non-viral gene carrier [163].

A carboxymethylated guar gum-grafted-polyethyleneimine copolymer (CMGG-g-PEI) was reported as an effective gene carrier in another study by Jana et al. [168]. The less toxic profile of CMGG-g-PEI was revealed by cytotoxicity and blood compatibility experiments. The CMGG-g-PEI/pDNA complex’s in vitro gene transfection efficiency was improved in A549 cells where CMGG-g-PEI showed higher transfection efficiency in comparison to the well-known conventional polymer, polyethyleneimine (PEI).

### 7.2. Applications of Plant-Based Mucilages in Nanomedicine

The growing interest in the use of natural components has led to strong attention to the scientific and medical use of plants for a variety of applications [8,9,102,150]. Mucilages are polysaccharide hydrocolloids with significant physical, chemical, and structural variations and distinctive functional and medical benefits [22,32,169]. They are recognized for their antibacterial, antihypertensive, antioxidant, antiasthmatic, hypoglycemic, and hypolipidemic actions and, also, their function as linking, thinning, sustaining, and humidification agents. In addition to offering advantages for delivery properties, the encapsulation of food ingredients, pharmaceutical, and nutraceutical materials is an important prospect to increase the stabilization of bioactive compounds [170].

Drug delivery is one of the promising applications for mucilage [170]. The development of new drug delivery platforms using both synthetic (like PE, PP, and PDMS) and natural polymers has been widely studied in recent years [171]. After all, the use of polymers of natural origin (plant-derived polymers) for pharmaceutical formulations is more desirable and is identified as a crucial factor in the development of improved drug delivery systems [23]. In contrast to synthetic polymers, natural polymers are less toxic, as well as biocompatible, sustainable, cheap, readily available, biodegradable, and reusable, and have the capacity for functional alterations [172]. In a study conducted by Ghoreishi et al., for example, paclitaxel (PX), a potent anticancer drug, was loaded into basil seed mucilage (BSM) aerogels by use of supercritical carbon dioxide (SC-CO_2_) processing [173]. The influence of system parameters on the mean particle size of PX, particle size distribution, and encapsulation efficiency (EE %) were studied. Paclitaxel NPs had a size of 82–131 nm, narrow size distribution, and EE% of 28–52%. Also, data showed that bigger dimethyl sulfoxide (DMSO)/water ratio, concentration of ethanol, and pressure and rate of CO_2_ addition can decrease size and EE%.

Another application of mucilage is cell proliferation scaffolds. In recent research, electrospun nanofibers (ESNFs) were produced from mucilage isolated from chan and linaza beans and mozote stem available commercially in Costa Rica, as reported by Hilary Urena-Saborio [174]. As an assisting component, poly(vinyl alcohol) (PVA) was applied. Findings demonstrated that plant mucilage-based ESNFs were well-suited for the growth of fibroblast cells, considerably better than PVA ESNFs; and chan bean mucilage was more effective for promoting cell proliferation than mozote and linaza.

In past years, the use of mucilages in wound healing has risen dramatically [175]. In Iranian traditional medicine, quince seed mucilage (QSM) has been used for the treatment of wounds and burns. Recent studies suggest that QSM has improved the healing of wounds [175]. Tamri et al. investigated the therapeutic capacity of QSM formulated as 5%, 10%, and 20% eucerin-based creams, with particular attention to growth factors that require tissue repair. Findings demonstrated that, on most days, there were statistically important differences in wound contraction between QSM 10 and 20% cream care and control groups (*p* < 0.05). The best outcomes were rabbits treated with QSM 20 percent cream (completed healing in 13 days, higher hydroxyproline content, and higher tissue resistance and higher wound fluid levels of evaluated growth factors). Goulart Carvalho et al. investigated the wound healing properties and mucilage content of *Pereskia aculeata* from various substrates in a related review [176]. The findings demonstrated that, despite the soil treatment obtained along with the different biomes where *P aculeata* is cultivated, the substrate used in cultivation may interfere with mucilage formation but not with cytotoxicity and wound healing, which demonstrates the protection of its use. Also, morphological studies have shown the favorable impact of the mucilage-containing extract on the culture of fibroblast cells, supporting its common use for tissue and wound regeneration.

**Figure 4:**
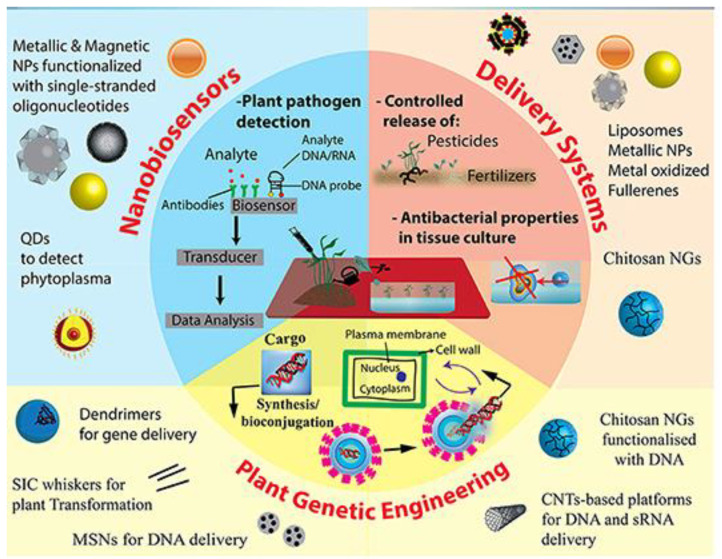
Different application of plant-based gums and mucilages in nanomedicine [177].

A schematic representation for different applications of plant-based gums and mucilages in nanomedicine is shown in Figure 4. Table 3 also shows the thematic classification of some applications of plant-based gums and mucilages in the field of nanoscience and nanotechnologies.

## 8. Challenges and Future Scope

While mucilages and gums are obtained from nature, their availability varies depending on the conditions of the climate and season. Extraction and purifying are essential processes after processing [188]. The growth and productivity of mucilages and gums may also be affected by morphological characteristics (such as seed coat), physical damage to seeds, and improper removal of mucilages and gums, presenting a significant obstacle to costs associated and the potential for a mass level of production. The equilibrium moisture level of mucilages and gums is about 10 percent and the possibility of microbiological pathogens at any point of its processing is probable. The storage conditions are another main operation; studies have confirmed differences in mucilage and gum quality with storing [32]. Of course, it is important to better explore restriction elements considering various applications of mucilages and gums in therapeutics, and investigations must be taken to explore the scope of mucilages and gums in terms of price, usability, functionality, and scalability compared to conventional encapsulation materials. Metallic nanoparticles extracted from plants are expected to have an impact on the diagnosis and treatment of different diseases with controlled side effects [124,189]. Furthermore, plants have a broad perspective on the preparation of metallic nanoparticles in healthcare and consumer goods.

## 9. Conclusions

Natural polysaccharides, particularly gums and mucilages, are considered sustainable materials due to their unique structural, biological, physicochemical, and biomechanical features. The gums and mucilages derived from plants are well known to science and are widely used in food-processing, pharmaceutics, and nanomedicine. Outstanding examples of these include gum tragacanth (from several species of the genus *Astragalus*), and gum arabic (*Acacia senegal* (L.) Willd.). These natural materials have advantages over synthetic ones owing to their outstanding structural features, less expensive price, nontoxicity, ease of modification, biocompatibility, abundant availability, and also promising potential. The materials of choice for different pharmaceutical applications are plant-based gums and mucilages, due to their biodegradability, adequate supply, low-toxicity, and simple processing conditions. Originally, gums and mucilages based on plants were used in formulations as an excipient to improve physical and chemical properties and stabilization. Plant-based gums and mucilages are used by many pharmaceutical formulations as one of their main ingredients to date. In novel drug and gene delivery systems, the task of plant-based gums and mucilages has affected the overall understanding of plant-based gums and mucilages and gained them an identity as a possible matrix/carrier material for a broad range of new drug delivery systems. A new type of polymeric material explored for pharmaceutical applications is modified plant-based gums and mucilages, which have extended the scope of gums in the production of formulations. In addition, some recent applications of plant-based gums and mucilages in the field of biosynthesis and gene delivery of nanotechnology create an alternative path for more study and applications of plant-based gums and mucilages in the development of novel drug delivery systems.

## Figures and Tables

**Table 1:** Some of the most important botanical sources of gums and their pharmaceutical applications.

Substance (Common Name)	Botanical Name	Family	Structure	Pharmaceutical Application	Ref
Guar gum	*Cyamopsis tetragonoloba* (L.) Taub.	Fabaceae	Galactose Mannose	Sustained release Controlled drug delivery Suspending agent	[34,35,36,37]
Almond Gum	*Prunus dulcis* (Mill.) D.A.Webb	Rosaceae	Aldobionic acid L-arabinose L-galactose D-mannose	Emulsifying Thickening Suspending Adhesive Stabilizing ↑Drug release Uncoated tablet dosage form	[38]
Karaya gum	*Firmiana simplex* (L.) W.Wight	Malvaceae	α-d-galacturonic acid α-l-rhamnose	In vivo → gastric retentive dosage forms ↑Dissolution rate of drug solid dispersions Suspending agent Emulsifying agent Dental adhesive Sustaining agent Mucoadhesive Buccoadhesive	[39]
Tragacanth gum	*Astragalus brachycalyx* Fisch. ex Boiss., *A. gummifer* Labill.	Fabaceae	Pectinaceous Arabino galactans Xylogalacturonans	Sustain release Suspending agent Emulsifying agent	[40,41,42]
Tamarind gum	*Tamarindus indica* L.	Fabaceae	Glucosyl: Xylosyl: Galactosyl 3:2:1	Matrix tablets ↓Drug release Biodegradable carrier for colon specific release	[23]
Grewia gum	*Grewia mollis* Juss.	Malvaceae	Glucose Rhamnose Galacturonic acid	Controlled release dosage forms Suspending agent Binding property ↑Degree of packing ↑Fluidity granules In vitro drug release → control the release of cimetidine from tablets delaying the release of cimetidine from tablets Film forming property	[43,44,45,46,47,48]
Gum acacia	*Acacia nilotica* (L.) Delile	Fabaceae	1,3-linked β-d-galactopyranosyl	Binder Suspending agent Emulsifying agent Demulcent Emollient	[49,50]
Khaya gum	*Khaya grandifoliola* C.DC.	Meliaceae	Protein Sugar Phenol 61% Galactose 14% Arabinose 7% Rhamnose, 8% Glucose 5% Glucuronic acid <2% other sugar residues	Binding agent Drug targeting Controlled release	[51,52]
Locust bean gum (carob gum)	*Ceratonia siliqua* L.	Fabaceae	D-galacto- Dmannoglycan pentane Proteins Cellulose	Super disintegrant Controlled drug delivery Drug targeting to the colon Super disintegrants Mucoadhesive	[53,54,55,56]
Terminalia catappa gum	*Terminalia catappa* L.	Combretaceae	_____	Oral sustained release tablets	[57]
Okra gum	*Abelmoschus esculentus* (L.) Moench	Malvaceae	Galactose Galacturonic acid Rhamnose Glucose Mmannose Arabinose Xylose	Controlled release tablet Sustained-release tablets Suspending agent	[58]
Gum ghatti	*Anogeissus latifolia* (Roxb. ex DC.) Wall. ex Guillem. and Perr.	Combretaceae	β-1-3-linked D galactose units with some ß1-6- linked D-galactose units	Binder Emulsifier Suspending agent	[59,60]
Albizia gum	*Albizia zygia* (DC.) J.F.Macbr.	Fabaceae	Galactose Mannose Arabinose Glucuronic acid 4-*0*-α-methyl analogue	Tablet binder Emulsifier Coating materials in compression-coated tablets	[61,62]
Cashew gum	*Anacardium occidentale* L.	Anacardiaceae	Galactose Arabinose Rhamnose Glucose Glucuronic acid L-arabinose L-rhamnose D-galactose Glucuronic acid	Suspending agent ↑Disintegration time ↑Polymer ratio → ↓drug release to a greater extent	[63,64,65]
Bhara gum	*Terminalia bellirica* (Gaertn.) Roxb.	Combretaceae	ß-sitosterol Gallic acid Ellagic acid Ethyl gallate Galloyl glucose Chebulaginic acid	Sustained release Microcapsules employing bhara gum → ↓release of famotidine	[66,67]
Cordia gum	*Cordia myxa* L.	Boraginaceae	Galactose (27%) Rhamnose (21%) Mannose (17%) Xylose (11%) Glucose (10%) Arabinose (9.5%) and uronic acids (5%)	Oral sustained release matrix tablets	[62]
Honey Locust Gum	*Gleditsia triacanthos* L.	Fabaceae	Proteins Fats Carbohydrates Fibers	Matrix tablets at different concentrations (5% and 10%)	[64,68]
Tara Gum	*Caesalpinia spinosa* (Molina) Kuntze	Fabaceae	Galactomannans. ratio of mannose to galactose in tara gumis 3:1	Controlled release carrier	[69,70]
Neem Gum	*Azadirachta indica* A.Juss.	Meliaceae	Mannose Glucosamine Arabinose Galactose Fucose Xylose Glucose	Binding property Sustained release ↑Matrix tablet	[71,72]
Moringa oleifera Gum	*Moringa oleifera* Lam.	Moringaceae	Arabinose Galactose Glucuronic acid in the preparation of 10:7:2 Rhamnose	Gelling property Binding property Release retardant property Disintegrating property Emulsifying property	[73,74,75,76]
Gum Damar	*Shorea javanica* Koord. and Valeton	Dipterocarpaceae	40% a Alpha resin (resin that dissolves in alcohol) 22% Beta-resin 23% Dammarol acid 2.5% Water	Sustained release	[77,78]
Hakea Gum	*Hakea gibbosa* Cav.	Proteaceae	Glucuronic acid Galactose Arabinose Mannose Xylose which is 12: 43: 32: 5: 8.	Sustained release Binding agent	[79,80,81]
Mango Gum	*Mangifera indica* L.	Anacardiaceae	______	Binding agent Sustained release Disintegrating	[82,83]
Olibanum Gum	*Boswellia serrata* Roxb. ex Colebr.	Burseraceae	5–9% Oil content 13–17% Resin acids, 20–30% Polysaccharides 40–60% boswellic acid	Sustained release Binding agent	[84,85]
Terminalia Gum	*Terminalia randii* Baker f.	Combretaceae	______	Binding agent ↑Strength friability ↓Friability	[86]
Konjac Glucomannan.	*Amorphophallus konjac* K.Koch	Araceae	D-glucose D-mannose in the ratio 1: 1.6	Gelling properties	[87]

**Table 2:** Some of the most important botanical sources of mucilage and their pharmaceutical applications.

Substance (Common Name)	Botanical Name	Family	Structure	Pharmaceutical Application	Ref
Mimosa mucilage	*Mimosa pudica* L.	Fabaceae	D-xylose, D-glucuronic acid	↓Release of drug from tablets In vitro release→↑mucilage ↓Release of drug ↑Mucilage in tablets→ ↑Percent swelling ↓Percent erosion of tablets	[115]
Hibiscus rosa-sinensis	*Hibiscus rosa-sinensis* L.	Malvaceae	L-rhamnose, D-galactose, D-galacturonic acid, D-glucuronic acid	Sustained release Binding agent Release-retarding agent	[116]
Asario Mucilage	*Lepidium sativum* L.	Brassicaceae	_______	Suspending agent Emulsifying agent	[21]
Fenugreek Mucilage	*Trigonella foenum-graecum* L.	Fabaceae	Mannose, Galactose, Xylose	Better release retardant	[117]
Aloe Mucilage	*Aloe vera* (L.) Burm.f.	Xanthorrhoeaceae	Arabinan, Arabinorhamnogalactan, Galactan, Galactogalacturan, Glucogalactomannan, Galactoglucoarabinomannan, Glucuronic acid, Polysaccharides	A controlled delivery system	[118]
Phoenix Mucilage	*Phoenix dactylifera* L.	Arecaceae	Carbohydrates 44–88%, Fructose, Sucrose, Mannose, Glucose, Maltose, Pectin (0.5–3.9%), Starch, Cellulose	Binding properties	[116]
Cassia tora Mucilage	*Senna tora* (L.) Roxb.	Fabaceae	Cinnamaldehyde, Tannins, Mannitol, Coumarins, Essential oils, (aldehydes, eugenol, pinene), Sugars, Resins	Binding Property ↑Hardness ↓Disintegration Suspending agent	[119]
Dendrophthoe Mucilage	*Dendrophthoe falcata* (L.f.) Ettingsh.	Loranthaceae		Binder	[120]
Cocculus Mucilage	*Cocculus hirsutus* (L.) W.Theob.	Menispermaceae	Polysaccharides, Gelatinous type of material	Gelling property Anti-inflammatory	[121]
Cordia Mucilage	*Cordia dichotoma* G.Forst.	Boraginaceae	______	Binding agent Emulsifying	[122]
Ocimum Mucilage	*Ocimum americanum* L.	Lamiaceae	Xylose, Arabinose, Rhamnose, Galacturonic acids	Disintegrating property	[123]

**Table 3:** Some applications of gums and mucilages in nanomedicine.

No	Genus and Used Form	Application	Results	Reference
1	Basil seed mucilage	Antimicrobial	basil seed mucilage–chitosan films containing *Ziziphora clinopodioides* essential oil and MgO nanoparticles can be used for increasing shelf-life of stored food commodities	[178]
2	Quince seed mucilage	cell culture scaffolds	the electrospun quince seed mucilage, in combination with polycaprolactone based scaffolds with 3D structures and 75–150 nm mean fiber diameters, are able to maximize adhesion and growth of epithelial Vero cells.	[179]
3	Quince seed mucilage	Structural improvement and antibacterial	quince seed mucilage supplemented with titanium dioxide (TiO_2_) and silicon oxide (SiO_2_) nanoparticles greatly improved the antibacterial and physico-mechanical properties of the prepared films.	[180]
4	Asafoetida gum	Cell toxicity and antimicrobial	Synthesized silver nanoparticles using Asafoetida were found to be effective in inhibiting the multiplication of cancer cells (MCF-7). They also exhibited significant antibacterial and antifungal activity.	[181]
5	*Alyssum homolocarpum* seed gum	Synthesis of magnetite nanoparticles and antibacterial	Magnetic nanocomposite (Fe_3_O_4_ NPs) was synthesized and coated via *Alyssum homo-locarpum* seed gum successfully. The fabricated nanocomposite exhibits excellent antibacterial activity against Gram-positive and Gram-negative bacteria.	[182]
6	Guar gum	Biosynthesis of nanocomposites and Agricultural industry	Novel (Carboxymethyl cellulose) CMC–guar gum silver nanocomposites (CG-Ag0NC) are fabricated. Antimicrobial results displayed greater performance of the CG-Ag0NC. Developed CG-Ag^0^NC enhanced the shelf life of strawberries.	[183]
7	*Azadirachta indica* gum	Nano-carrier	Gum purified from *Azadirachta indica* did not have antibacterial activity but possessed good antioxidant and anticancer activity. The extracted polysaccharide was further carboxymethylated and used for the synthesis of nanocarrier to carry anticancer drug, curcumin. The nanocarriers were found to be effective against MCF7 cancer cell line.	[184]
8	Guar gum	Water purification	Guar gum–nano zinc oxide (GG/nZnO) biocomposite was used as an adsorbent for enhanced removal of Cr(VI) from aqueous solution.	[185]
9	Persian gum	Food industry	Nano-capsules with fish oil–garlic essential oil using persian gum were successfully produced. Nano-capsules produced have good physicochemical properties indicating good stability.	[186]
10	Gum kondagogu	Removal of various toxic metal ions	Gum kondagogu (GK) modified magnetic iron oxide nanoparticles (MNP). The removal efficiencies for a variety of metal cations by the GK–MNP were determined quantitatively in the order: Cd^2+^ > Cu^2+^ > Pb^2+^ > Ni^2+^ > Zn^2+^ > Hg^2+^	[187]

## Data Availability

The data presented in this study are available upon request from the corresponding author.
